# Association Between Sclerostin and Sarcopenia-Related Functional Decline in Older Women

**DOI:** 10.3390/diagnostics16020272

**Published:** 2026-01-14

**Authors:** Dong Gyu Lee, Jong Ho Lee, Eunjung Kong

**Affiliations:** 1 Department of Physical Medicine and Rehabilitation, College of Medicine, Yeungnam University, Daegu 42415, Republic of Korea; 2 Department of Laboratory Medicine, College of Medicine, Yeungnam University, Daegu 42415, Republic of Korea; ae4207@naver.com; 3 Department of Nuclear Medicine, College of Medicine, Yeungnam University, Daegu 42415, Republic of Korea; kongej@yu.ac.kr

**Keywords:** sclerostin, sarcopenia, osteoporosis, bone mineral density, grip strength

## Abstract

**Background:** Sclerostin, an osteocyte-derived glycoprotein, plays a key role in bone metabolism by inhibiting the Wnt/β-catenin signaling pathway. While it is a recognized therapeutic target in osteoporosis, its relationship with sarcopenia remains unclear. This study aimed to investigate the associations between serum sclerostin levels, sarcopenia, and osteoporosis in older women. **Methods:** We conducted a cross-sectional study of 79 postmenopausal women aged ≥65 years. Sarcopenia was defined based on grip strength and appendicular skeletal muscle mass (ASM), osteoporosis was diagnosed according to femoral T-scores, and serum sclerostin levels were measured using ELISA. Associations with clinical variables and bone mineral density (BMD) were evaluated using correlation and logistic regression analyses. **Results:** Sclerostin levels were significantly higher in women with sarcopenia (*p* = 0.036) and exhibited a negative correlation with grip strength (r = −0.298, *p* = 0.008) but not with ASM. Positive correlations were found between sclerostin and multiple femoral BMD parameters. In a logistic regression analysis, sclerostin was modestly associated with sarcopenia (*p* = 0.045); however, no significant association was observed with osteoporosis (*p* = 0.257). **Conclusions:** Elevated sclerostin levels are associated with reduced muscle strength and sarcopenia in older women, independent of muscle mass, indicating that sclerostin may reflect a functional decline in musculoskeletal health. Muscle strength should therefore be considered when interpreting sclerostin’s clinical implications in aging populations.

## 1. Introduction

Sarcopenia and osteoporosis are two of the most common musculoskeletal conditions that affect older adults [1]. The former is defined by a gradual decline in muscle mass and strength, leading to reduced mobility, an increased risk of falls, and the loss of independence [2], while the latter is marked by reduced bone density and a deterioration of bone quality, significantly increasing the risk of fractures [3]. Although these conditions have traditionally been studied separately, accumulating evidence suggests they share overlapping biological mechanisms [4]. This has led to the recognition of a combined condition known as osteosarcopenia [5].

Bone and muscle are not only structurally interlinked but also participate in active biochemical communication. Recent studies have highlighted the roles of various signaling molecules—such as myokines, osteokines, and inflammatory mediators—in mediating this bidirectional interaction, thereby influencing the maintenance and function of both [6,7]. Among them is sclerostin, a glycoprotein predominantly secreted by osteocytes; it is known to inhibit the Wnt/β-catenin signaling pathway, playing a critical role in suppressing bone formation [8,9]. Osteocytes function as mechanoreceptors that sense mechanical strain within the bone microenvironment and regulate the secretion of sclerostin accordingly [10]. This mechano-sensation suggests that muscle-derived mechanical loading can modulate sclerostin expression [11].

Previous studies suggest that reduced physical activity may increase circulating sclerostin levels [12]. In patients with spinal cord injuries, sclerostin levels are elevated in the early stages of disuse, highlighting the importance of mechanical loading in its regulation [13,14]. Consequently, reduced physical activity associated with muscle weakness or sarcopenia may result in elevated sclerostin levels and impaired bone metabolism [15]. In older adults with diminished mobility, this link between sarcopenia and osteoporosis may become more pronounced [16].

Romosozumab, a monoclonal antibody targeting sclerostin, has shown clinical efficacy in improving bone mineral density (BMD) and reducing fracture risk in patients with osteoporosis [17]. However, the role of sclerostin in muscle metabolism is still unclear.

Given that sarcopenia leads to reduced physical activity [18], it could secondarily increase the expression of sclerostin [19], potentially affecting both bone and muscle. While the effects of sclerostin on bone formation are well understood, its association with muscle mass and function requires further study. Therefore, exploring the relationship between circulating sclerostin levels and bone health in this population could yield important insights into the shared pathophysiological mechanisms of these conditions and inform targeted therapeutic interventions.

Studying women aged 65 and older can be useful in clarifying the relationship between sclerostin and both bone and muscle health. After menopause, estrogen levels drop quickly—crucial, considering the key role this hormone plays in maintaining bone density and muscle mass [20]. As a result, postmenopausal women are more likely to experience both bone loss and muscle weakening, increasing the risk of osteosarcopenia. By focusing on this population, this study avoids variability from sex hormone differences and further elucidates how sclerostin is involved.

This study aimed to investigate the association between circulating sclerostin levels and the coexistence of sarcopenia and osteoporosis in community-dwelling older women. Given that sclerostin levels tend to increase with age, comparisons between younger and older individuals may be confounded by age-related physiological changes. By focusing solely on older women—including those with pronounced sarcopenia—this study minimizes the influence of age-related variability and provides a more targeted assessment of how muscle loss is associated with sclerostin concentrations. This research design addresses a specific gap in the current literature by clarifying the link between sarcopenia-related muscle weakness and circulating sclerostin within a high-risk, postmenopausal population.

## 2. Materials and Methods

### 2.1. Study Population

This cross-sectional analysis involved 79 community-dwelling women aged 65 years or older who visited the spine outpatient clinic of a university-affiliated hospital between February 2023 and November 2024. Participants were prospectively enrolled. To be eligible, they had to be 65 years or older and have undergone DEXA scanning for a clinical assessment of bone density and body composition. The exclusion criteria for participants were as follows: prior femoral surgery; treatment for osteoporosis in the past 12 months; receiving dialysis; active malignancy with ongoing chemotherapy; or major neurological or musculoskeletal impairments that significantly limited mobility. Ethical approval was granted by the hospital’s Institutional Review Board (IRB No. YUMC 2022-10-050), and informed consent was obtained from all participants.

### 2.2. Data Collection and Assessments

Anthropometric data, including height and body weight, were used to calculate BMI scores (kg/m^2^). Bone mineral density (BMD) was evaluated using dual-energy X-ray absorptiometry (DEXA, Hologic Discovery Wi, Marlborough, MA, USA), measuring both right and left femoral neck and total femur. BMD values were expressed in g/cm^2^, and corresponding T-scores were also recorded.

Muscle mass was evaluated using DEXA-derived appendicular skeletal muscle mass (ASM), calculated by summing the lean mass of the arms and legs and dividing by height squared (kg/m^2^). This method is consistent with established practices in sarcopenia research and has been validated in previous population studies [21]. ASM cut-off values were based on thresholds defined by the Korean Working Group on Sarcopenia (KWGS), whereby ASM index values below 5.4 kg/m^2^ indicate low muscle mass in women [22]. Handgrip strength was measured using a digital handheld dynamometer (Jamar Plus+, Patterson Medical, Warrenville, IL, USA), with two measurements taken from the dominant hand and the average value used for analysis. This measurement method aligns with previously validated protocols in musculoskeletal research and has demonstrated reliability in community-based assessments of older adults [23]. The same investigator conducted the handgrip strength test using an identical protocol applied in prior related studies by the research team, thereby ensuring methodological consistency and reproducibility.

Blood samples were drawn from participants between 9:00 AM and 11:00 AM. Serum sclerostin concentrations were measured using the Human SOST Immunoassay ELISA kit (R&D Systems, Minneapolis, MN, USA), and all assays were performed in duplicate, adhering to the manufacturer’s protocol. Absorbance was read using the SpectraMax 190 microplate reader (Molecular Devices, Sunnyvale, CA, USA), and the intra-assay coefficient of variation (CV) was <5% and inter-assay CV < 7%. For the majority of the participants, the DEXA scan, handgrip strength measurement, and blood sampling were performed on the same day.

### 2.3. Diagnostic Criteria

Sarcopenia was diagnosed according to the Korean Working Group on Sarcopenia (KWGS) criteria, where both reduced muscle strength (handgrip strength below 18 kg) and low muscle mass (ASM index below 5.4 kg/m^2^) characterize the condition [22]. Osteoporosis was diagnosed based on a T-score below −2.5 at any femoral site, including the right or left femoral neck and total femur, in accordance with the WHO criteria [24].

### 2.4. Statistical Analysis and Sample Size

Descriptive statistics were applied to present baseline clinical characteristics, expressed as mean ± standard deviation for continuous variables. The Shapiro–Wilk test was employed to assess normality, and depending on the distribution, group comparisons between sarcopenia and non-sarcopenia groups were performed using either an independent *t*-test or the Mann–Whitney U test. Associations between sclerostin levels and clinical parameters—including grip strength and BMD—were evaluated using Pearson correlation coefficients. Binomial logistic regression analyses were conducted to investigate whether serum sclerostin independently predicted sarcopenia or osteoporosis.

Statistical analyses were performed using SPSS version 27, with a significance threshold of *p* < 0.05. The sample size of 79 was selected based on considerations of statistical power, ensuring sufficient sensitivity to detect clinically meaningful differences. With this number of participants, this study was calculated to have more than 80% power to identify a medium effect size (Cohen’s d ≈ 0.5) in group comparisons and detect correlations of at least 0.3, using a two-sided alpha of 0.05. Although modest in size, the study population consisted exclusively of women aged 65 years and older, contributing to sample homogeneity and enhancing internal consistency for evaluating age-related musculoskeletal outcomes.

## 3. Results

Among the 79 participants, 39 (49.4%) were classified as having sarcopenia based on established diagnostic criteria (Table 1). Compared to the non-sarcopenic group, individuals with sarcopenia were significantly older (80.1 ± 5.32 vs. 77.2 ± 5.76 years, *p* = 0.015) and had lower BMI scores (23.2 ± 4.05 vs. 25.0 ± 2.90 kg/m^2^, *p* = 0.018), reduced ASM (4.85 ± 0.45 vs. 5.33 ± 0.87 kg/m^2^, *p* < 0.001), and lower grip strength (14.4 ± 3.19 vs. 20.0 ± 4.70 kg, *p* < 0.001). Serum sclerostin levels were significantly elevated in the sarcopenia group (158 ± 52.4 vs. 126 ± 75.7 pg/mL, *p* = 0.036), and regarding BMD, no significant group differences were observed for most regions.

**Figure 1:**
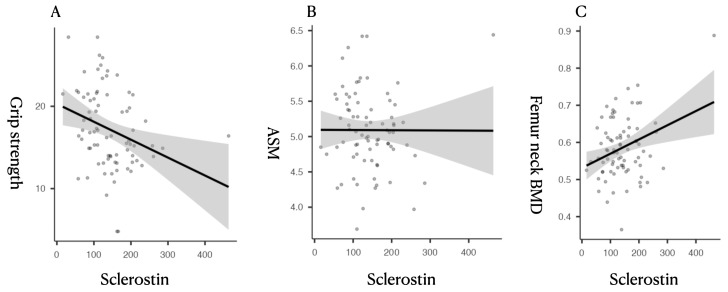
Scatterplots showing the relationship between circulating sclerostin levels and sarcopenia-related parameters. (**A**) Sclerostin vs. Grip Strength: A significant inverse correlation was observed between serum sclerostin and handgrip strength (*r* = −0.298, *p* = 0.008), suggesting that higher sclerostin levels are associated with lower muscle function. (**B**) Sclerostin vs. ASM (Appendicular Skeletal Muscle Index): No significant correlation was found between sclerostin and ASM (*r* = −0.003, *p* = 0.0976), indicating its limited association with muscle quantity. (**C**) Sclerostin vs. Femoral Neck BMD (g/cm^2^): A significant positive correlation was found between sclerostin and femoral bone mineral density (*r* = 0.391, *p* < 0.001), supporting its established role in bone metabolism.

Correlation analysis revealed that serum sclerostin was inversely correlated with grip strength (*r* = −0.298, *p* = 0.008) and positively correlated with femoral BMD parameters, including right femur neck BMD (*r* = 0.313, *p* = 0.005), total BMD (*r* = 0.391, *p* < 0.001), and respective T-scores (Table 2, Figure 1). No significant correlations were found between sclerostin and ASM, BMI, or age.

Univariate logistic regression analysis revealed that serum sclerostin was significantly associated with sarcopenia (estimate = 0.008, *p* = 0.045) but not osteoporosis (*p* = 0.257), suggesting its stronger relationship with muscle function than bone fragility (Table 3). When applying an ROC-derived cutoff value (serum sclerostin ≥ 124.6 pg/mL), individuals with higher sclerostin levels showed significantly increased odds of sarcopenia (OR = 5.39). However, the overall discriminative performance of serum sclerostin was modest, as continuous sclerostin levels showed limited predictive ability, and cutoff-based analysis provided only a weak improvement in discrimination.

## 4. Discussion

This study explored the relationship between circulating sclerostin levels and two age-related musculoskeletal conditions—sarcopenia and osteoporosis—in postmenopausal women. We found that higher serum sclerostin levels were significantly associated with the presence of sarcopenia, particularly through an inverse correlation with grip strength, whereas no such association was observed with osteoporosis. These results support the established role of sclerostin in bone metabolism and suggest that muscle function may also influence its regulation.

Primarily secreted by osteocytes, sclerostin inhibits bone formation by binding to LRP5/6 receptors on osteoblasts, thus antagonizing the Wnt/β-catenin signaling pathway [25,26,27]. Its expression is regulated via mechanical loading: osteocytes function as mechano-sensors that modulate the secretion of sclerostin in response to strain [28,29]. In addition to its inhibitory effects on the differentiation and activity of osteoblasts, sclerostin suppresses the expression of Runx2 and reduces the activity of alkaline phosphatase [30], both of which are essential for the formation of bone matrices. Circulating sclerostin levels have been observed to increase with age, potentially reflecting declining mechanical stimuli and cumulative skeletal unloading [31]. Moreover, sclerostin can indirectly inhibit bone resorption by modulating RANKL/OPG balance, further influencing bone remodeling dynamics [32]. As physical activity declines in aging individuals with sarcopenia, this reduced loading likely contributes to elevated sclerostin levels, impairing bone remodeling and exacerbating musculoskeletal decline.

Characterized by the age-related loss of muscle mass and strength, sarcopenia contributes to functional decline and an increased risk of falls and fractures. One of its main effects is diminished muscle-derived mechanical loading, which disrupts bone remodeling signals [33]. In our analysis, sclerostin levels were significantly elevated in individuals with sarcopenia. Notably, sclerostin was inversely correlated with grip strength, a functional indicator of muscle performance, but not with ASM. This finding suggests that the secretion of sclerostin is more sensitive to changes in muscle function and mechanical load than to static measurements of muscle quantity. According to the consensus definition by the European Working Group on Sarcopenia in Older People (EWGSOP), low muscle strength is the primary criterion for the diagnosis of sarcopenia [34], with ASM serving as a confirmatory indicator rather than a standalone diagnostic feature. Our study demonstrated that circulating sclerostin levels are more closely associated with muscle strength than muscle mass, suggesting its function as a potential biomarker for monitoring muscle function in individuals with sarcopenia.

Although experimental studies have consistently shown that increased sclerostin levels suppress bone formation, clinical findings in humans have been less consistent. Gorter et al. reported that patients with osteoporotic fractures exhibited lower sclerostin levels, indicating reduced osteocyte function in advanced bone fragility [35]. Conversely, Yamamoto et al. observed that higher sclerostin levels were associated with a greater risk of vertebral fracture in individuals with type 2 diabetes [36]. Moreover, despite its inhibitory role in bone formation, serum sclerostin has been found to be positively correlated with BMD in osteoporotic individuals, potentially due to a reduced number of osteocytes in severely osteoporotic bone [37]. These conflicting results may reflect differences in comorbidities, skeletal sites, or overlooked physiological mediators such as muscle strength. In our study, while body weight did not significantly correlate with sclerostin, it was independently associated with sarcopenia. Given that a loss of muscle mass is characteristic of sarcopenia, reductions in body weight may not be a truly independent predictor but rather a consequence of the syndrome itself, potentially introducing analytical bias.

Traditionally regarded as a bone-specific regulator secreted by osteocytes, sclerostin is now recognized as a multifunctional molecule with expression reported in skeletal muscle, cartilage, kidney, liver, and the cardiovascular and immune systems [38]. Recent studies have even identified the expression of sclerostin in muscle tissue, supporting its emerging classification as a myokine. This suggests its participation in bone–muscle endocrine crosstalk, particularly relevant in sarcopenia, where impaired muscle-derived mechanical and biochemical signals may influence sclerostin-mediated bone remodeling. Therefore, overlooking muscle function when interpreting serum sclerostin levels may lead to an incomplete understanding of its clinical implications in age-related musculoskeletal decline.

According to our logistic regression analysis, while sclerostin was significantly associated with sarcopenia, it was not a significant predictor of osteoporosis. This demonstrates that sclerostin alone may not be a sufficient marker of osteoporosis. Rather, its clinical relevance may lie in its connection to functional musculoskeletal decline. These findings highlight the need to integrate muscle performance metrics when assessing the role of sclerostin in bone health. Although our study confirmed that sclerostin correlates with BMD, its predictive value for osteoporosis may depend on broader physiological contexts, including comorbid conditions and physical activity status.

We restricted our study to postmenopausal women aged 65 years and older to reduce variability related to sex hormones and to focus on a population at high risk for both muscle and bone loss. The abrupt estrogen decline after menopause contributes to the deterioration of both tissues, making this group particularly relevant for studying osteosarcopenia.

This study has several limitations that must be acknowledged. As it was cross-sectional in nature, we could not determine whether increased sclerostin levels preceded or followed musculoskeletal decline. The modest sample size may also limit the generalizability of the findings, particularly for subgroup analyses. Although our power calculation was based on previously reported effect sizes for sclerostin–grip strength correlations, the lack of adjustment for multiple rounds of testing may have increased the risk of a Type I error. Moreover, while the exclusion criteria addressed some medical conditions, residual confounding due to unmeasured factors—such as physical activity levels, nutritional status, years since menopause, and the use of medications including hormone replacement therapy—may have influenced the results [39]. Moreover, detailed information on concomitant medications or supplements known to affect bone or muscle metabolism—such as vitamin D or calcium supplementation, statins, corticosteroids, or hormone-related therapies—was not systematically collected in this study. In addition, we did not account for comorbidities such as chronic kidney disease or metabolic syndrome, which are known to alter sclerostin levels. Finally, no correction for the inter-assay variability of the ELISA kit was presented, limiting the reproducibility of our results.

Despite these limitations, the findings suggest that sclerostin may reflect the interplay between declining muscle function and bone remodeling. It has potential as a shared biomarker—and possibly a therapeutic target—in age-related musculoskeletal decline. Our results underscore the need for integrated assessment strategies targeting both muscle and bone in older adults. Future research should aim to investigate longitudinal changes in sclerostin levels with aging and their temporal relationship with the progression of sarcopenia, incorporating physical activity metrics and stratified analysis by comorbidity profiles.

## 5. Conclusions

This study demonstrates that serum sclerostin levels are significantly associated with sarcopenia but not with osteoporosis or vertebral fracture in postmenopausal women. The observed inverse correlation with grip strength highlights the importance of muscle function in sclerostin regulation. These results indicate that sclerostin may serve as a marker of age-related musculoskeletal decline, particularly in the context of functional impairment. Given its established role in bone biology and emerging links with muscle strength, sclerostin represents a potential biomarker for osteosarcopenia. Future longitudinal studies are warranted to clarify its causal role and therapeutic potential in the management of concurrent muscle and bone loss. Future studies should also investigate the relationships between sclerostin and key factors influencing bone and muscle health, including physical activity and nutritional status. These factors may be assessed using smartphone-based activity tracking data or biochemical markers such as serum protein or albumin levels and glycated hemoglobin (HbA1c). Exploring how these variables relate to sclerostin and muscle volumetric indices could provide a clearer understanding of the observed associations and support the potential clinical use of sclerostin as a biomarker of muscle function.

## Figures and Tables

**Table 1:** Group comparisons of clinical parameters.

Variable			Non-Sarcopenia (40) (Mean ± SD)	Sarcopenia (39) (Mean ± SD)	*p*-Value
Age			77.2 ± 5.76	80.1 ± 5.32	0.015 *
BMI			25.0 ± 2.90	23.2 ± 4.05	0.018 *
ASM			5.33 ± 0.87	4.85 ± 0.45	<0.001 ***
Grip Strength			20.0 ± 4.70	14.4 ± 3.19	<0.001 ***
Sclerostin (pg/mL)			126 ± 75.7	158 ± 52.4	0.036 *
Femur BMD	Neck	Rt. (g/cm^2^)	0.599 ± 0.084	0.572 ± 0.077	0.364
		Lt. (g/cm^2^)	0.611 ± 0.108	0.567 ± 0.089	0.097
		Rt. T-score	−1.92 ± 0.795	−2.16 ± 0.716	0.379
		Lt. T-score	−1.81 ± 1.03	−2.29 ± 0.665	0.065
	Total	Rt. (g/cm^2^)	0.724 ± 0.103	0.709 ± 0.102	0.922
		Lt. (g/cm^2^)	0.734 ± 0.112	0.706 ± 0.088	0.364
		Rt. T-score	−1.14 ± 0.933	−1.24 ± 0.889	0.980
		Lt. T-score	−1.01 ± 0.984	−1.26 ± 0.775	0.393

Skeletal muscle mass. Statistical tests: Mann–Whitney U test. * *p* < 0.05, and *** *p* < 0.001. Abbreviations: BMI = body mass index; ASM = appendicular skeletal muscle mass; and BMD = bone mineral density.

**Table 2:** Correlation matrix between sclerostin and clinical/BMD parameters.

Variable				Pearson’s *r*	*p*-Value
Age				0.208	0.065
BMI (kg/m^2^)				0.088	0.440
Grip Strength (kg)				−0.298 **	0.008 *
ASM (kg/m^2^)				−0.003	0.976
Femur BMD	Neck	BMD (g/cm^2^)	Rt.	0.313 **	0.005 *
			Lt.	0.185	0.102
		T-score	Rt.	0.308 **	0.006 *
			Lt.	0.221	0.051
	Total	BMD (g/cm^2^)	Rt.	0.391 ***	<0.001 ***
			Lt.	0.342 **	0.002 *
		T-score	Rt.	0.381 ***	<0.001 ***
			Lt.	0.342 **	0.002 *

Note: Pearson correlation coefficients for serum sclerostin and clinical/BMD parameters. * *p* < 0.05, ** *p* < 0.01, and *** p < 0.001. Abbreviations: ASM = appendicular skeletal muscle mass; BMD = bone mineral density; and BMI = body mass index.

**Table 3:** Association of serum sclerostin with sarcopenia and osteoporosis: Univariable logistic regression analyses using continuous serum sclerostin levels and ROC-derived cutoff values.

Outcome	Analysis Type	Cut-Off (pg/mL)	OR	95% CI	*p*-Value
Sarcopenia	Continuous		1.008	1.000–1.020	0.045 *
	Cut-off (≥124.6)	≥124.6	5.39	2.04–14.19	<0.001 *
Osteoporosis	Continuous		1.004	0.998–1.010	0.257
	Cut-off (≥83.2)	≥83.2	0.26	0.007–0.96	0.043 *

Note: Continuous analyses reflect the change in odds per 1 pg/mL increase in serum sclerostin. Cutoff values were determined using the Youden index derived from receiver operating characteristic (ROC) analysis for each outcome. Odds ratios (ORs) are presented with 95% confidence intervals (CIs). * *p* < 0.05.

## Data Availability

The data presented in this study are available on request from the corresponding author due to ethical restrictions and privacy concerns related to human participant data.

## Abbreviations

The following abbreviations are used in this manuscript:

ASM	Appendicular Skeletal Muscle Mass
BMD	Bone Mineral Density
BMI	Body Mass Index
DEXA	Dual-Energy X-ray Absorptiometry
ELISA	Enzyme-Linked Immunosorbent Assay
KWGS	Korean Working Group on Sarcopenia
